# Supplementary material for: Collective dynamics support group drumming, reduce variability, and stabilize tempo drift
Source: eLife. 2022 Nov 1;11:e74816. doi: 10.7554/eLife.74816 (PMC9678363; doi:10.7554/eLife.74816)
Supplement: Supplementary file 1. — Separate linear mixed-effects models were fitted for each of lags 1–8 of the autocorrelations of individuals (a) and group aggregates (b) in the continuation phases of synchronization-continuation task (SCT) trials, ensemble conditions. The predictors were group size (N) and Tempo (80, 120, 160, and 200 beats per minute [bpm]). For simplicity, the same full model was fitted in all cases for individuals, Yik=(β0+σ0g)+β1Nik+β2Tempoik+β3NikTempoik+σg, where i is participant, k trial, and g group. For the group aggregate (b) where there was not enough data to fit models with all predictors, Yik=(β0+σ0g)+β1Nik+β2Tempoik+σg was used. Significant coefficients (p<.05, Satterthwaite method) are in bold. [file elife-74816-supp1.docx]

Supplementary Table. Separate linear mixed-effects models were fitted for each of lags 1 to 8 of the auto-correlations of individuals (a) and group-aggregates (b) in the continuation phases of SCT trials, ensemble conditions. The predictors were *group size* (*N*) and *Tempo* (80, 120, 160, 200 bpm). For simplicity, the same full model was fitted in all cases for individuals, *Y_ik_*=(*β_0_*+σ*_0g_*)+*β_1_N_ik_*+*β_2_Tempo_ik_*+*β_3_N_ik_Tempo_ik_*+σ*_g_*, where *i* is participant, *k* trial, and *g* group. For the group-aggregate (b) where there was not enough data to fit models with all predictors, *Y_ik_*=(*β_0_*+σ*_0g_*)+*β_1_N_ik_*+σ*_g_* was used. Significant coefficients (*p*<.05, Satterthwaite method) are in bold.

| Lag | 1 | 2 | 3 | 4 | 5 | 6 | 7 | 8 |
| --- | --- | --- | --- | --- | --- | --- | --- | --- |
| Parameters | *β*/*SE*/*t* | *β/SE/t* | *β/SE/t* | *β/SE/t* | *β/SE/t* | *β/SE/t* | *β/SE/t* | *β/SE/t* |
| a) Individuals | |  |  |  |  |  |  |  |
| β_0_: Intercept | **-.425/.028/-15.19** | -.028/.023/-1.25 | *t*<1 | **.060/.019/3.14** | **-.05/.019/-2.65** | .028/.017/1.63 | **-.058/.018/-3.22** | *t*<1 |
| β_1_: *N* | **.014/.005/3.01** | **-.012/.003/-4.46** | *t*<1 | **-.008/.002/-3.48** | *t*<1 | **-.004/.002/-1.99** | *t*<1 | **-.0066/.0029/-2.24** |
| β_2_: Tempo | **.001/.0001/6.29** | .0002/.0001/1.64 | **-.0004/.0001/-3.83** | *t*<1 | *t*<1 | *t*<1 | **.0002/.0001/1.99** | **.0004/.0001/4.10** |
| b) Group-aggregate | |  |  |  |  |  |  |  |
| β_0_: Intercept | **-.228/.022/-10.34** | **.053/.016/3.34** | -.032/.020/-1.64 | **.11/.016/6.68** | **-.044/.015/-2.98** | **.033/.014/2.38** | *t*<1 | **.088/.015/5.81** |
| β_1_: *N* | .0095/.0057/1.66 | *-*.005/.004/-1.18 | *t*<1 | **-.008/.004/-2.07** | .005/.004/1.20 | *t*<1 | *t*<1 | **-.009/.004/-2.38** |
|  |  |  |  |  |  |  |  |  |
